# Immunogenicity and safety of a split-virion quadrivalent influenza vaccine in adults 18–60 years of age in the Republic of Korea

**DOI:** 10.1080/21645515.2017.1381808

**Published:** 2017-11-17

**Authors:** Won Suk Choi, Ji Yun Noh, Jacob Lee, Jun Yong Choi, Jin-Soo Lee, Moo Soo Kim, Hee Soo Kim, Joon Bang, Nathalie Lavis, Woo Joo Kim

**Affiliations:** a Division of Infectious Diseases, Department of Internal Medicine, Korea University College of Medicine, Seoul, Republic of Korea; b Division of Infectious Diseases, Department of Internal Medicine, Hallym University College of Medicine, Seoul, Republic of Korea; c Division of Infectious Diseases, Department of Internal Medicine, Yonsei University College of Medicine, Seoul, Republic of Korea; d Division of Infectious Diseases, Department of Internal Medicine, Inha University School of Medicine, Incheon, Republic of Korea; e Sanofi Pasteur, Regulatory Affairs, Seoul, Republic of Korea; f Sanofi Pasteur, Global Medical Affairs, Seoul, Republic of Korea; g Sanofi Pasteur, Medical Operations, Lyon, France

**Keywords:** quadrivalent influenza vaccine, inactivated influenza vaccine, adults, Republic of Korea

## Abstract

VaxigripTetra® (Sanofi Pasteur, Lyon, France) is a quadrivalent split-virion inactivated influenza vaccine (IIV4) containing two B-lineage strains approved in the European Union and Taiwan in 2016 for individuals ≥ 3 years of age. Here, we describe an observer-blind, randomized, controlled, multicenter trial study evaluating the immunogenicity and safety of the Northern Hemisphere 2015–2016 formulations of IIV4 and the licensed split-virion trivalent inactivated influenza vaccine (IIV3) in the Republic of Korea (ClinicalTrials.gov no. NCT02550197). The study included 300 Korean adults 18–60 years of age randomized 2:1 to receive a single injection of IIV4 or IIV3. For each of the four vaccine strains in IIV4, 21 days after vaccination, geometric mean post-/pre-vaccination ratios of hemagglutination inhibition titers were ≥ 3.97. Seroconversion/significant increases rates were ≥ 40% for all but the A/H1N1 strain, for which the rate was 39.7%. Results were similar for the three strains in IIV3. For the additional B-lineage strain not in IIV3 (Victoria), hemagglutination inhibition antibody titers were higher for IIV4 than for IIV3. Solicited reactions and adverse events were similar between IIV4 and IIV3, and no serious adverse events or new safety signals were detected. These results confirm the robust immunogenicity and acceptable safety of IIV4 in adults 18–60 years of age and show that including a second B-lineage strain should provide broader protection against B-strain influenza without affecting vaccine safety or the immunogenicity of other three vaccine strains.

## Introduction

As in other countries, seasonal influenza is associated with significant morbidity and mortality in the Republic of Korea. Between 2003 and 2013, influenza was associated with an average of 2900 excess deaths per year, most of which were in older people.^1^ The total socioeconomic burden of seasonal influenza in the Republic of Korea was estimated at US $44.7 million in 2007–2008 and US $42.3 million in 2008–2009.^2^ Since 1997, the National Immunization Program has included vaccination against seasonal influenza as a supplementary activity.^3^ Initial recommendations were to vaccinate seniors, healthcare workers, persons with conditions that put them at high risk for influenza complications, and family members of persons at high risk for influenza complications.^4^ The recommendations have been expanded to include children 6–59 months of age, adults 50–64 years of age, persons with neuromuscular diseases, primary responders to avian influenza, and persons working on chicken, pig, or duck farms.^4^ Although influenza vaccination coverage rates are around 80% for older adults, the rates are only around 35% for adults 45–64 years of age and 25% for adults 19–44 years of age.^5^

In the mid-1980s, two distinct genetic lineages of influenza B virus, Victoria and Yamagata, emerged and, since the 2000s, they have been co-circulating worldwide.^6^ However, until recently influenza vaccines have included only a single B-lineage strain. Selecting the correct B-lineage strain for each year's formulation of trivalent influenza vaccine has been difficult.^7-9^ To help address co-circulation of the two B-strains lineages, trivalent influenza vaccines, which contain a single B-lineage strain, are gradually being replaced by quadrivalent vaccines containing B strains from both lineages.^9^

VaxigripTetra® (Sanofi Pasteur, Lyon, France) is a quadrivalent split-virion inactivated influenza vaccine (IIV4) approved in the European Union and Taiwan in 2016 for individuals 3 years of age and older. Phase III clinical trials^10-12^ have shown that IIV4 has been as immunogenic as the comparator split-virion trivalent inactivated influenza vaccine (IIV3; Vaxigrip®, Sanofi Pasteur) for each of the three shared strains and superior for the additional B strain. At the same time, safety profiles have been similar. In other words, adding a second B-strain lineage to IIV3 is expected to provide added coverage against influenza without affecting immune responses against the original three strains and without affecting safety. Here, we describe a study to confirm the immunogenicity and safety of the Northern Hemisphere 2015–2016 formulation of IIV4 in Korean adults 18–60 years of age.

## Results

### Participants

The study included 300 participants 18–60 years of age enrolled at five centers in the Republic of Korea between September 11, 2015 and November 2, 2015. The study was completed on November 26, 2015. The participants were vaccinated to receive a single injection of IIV4 (n = 200) or IIV3 (n = 100). All participants were vaccinated as randomized, and all completed the study. All were Asian, and mean ages and sex ratios were similar for the two vaccine groups (35.9 ± 9.1 years, 75% female for IIV4 and 35.6 ± 9.5 years, 71% female for IIV3). Vaccination with the seasonal influenza vaccines during the previous (2014–2015) influenza season was reported by 42.5% of participants in the IIV4 group and 33.0% in the IIV3 group. One participant (1.0%) in the IIV3 group reported a history of influenza illness during the previous (2014–2015) influenza season.

### Immunogenicity

IIV4 was evaluated in this study according to the former Committee for Human Medicinal Products (CHMP) Note for Guidance^13^ in agreement with the Republic of Korea Ministry of Food and Drug Safety. In the IIV4 group, most participants (82.9%–99.5%) had detectable baseline hemagglutination inhibition (HAI) titers (≥ 10) for all four vaccine strains (A/H1N1, A/H3N2, B Yamagata lineage, and B Victoria lineage). Seroprotection rates at baseline were at least 56.3% (A/H3N2) and as high as 91.5% (B Yamagata lineage) (Table 1). Despite the relatively high baseline immunogenicity against the vaccine strains, 21 days after vaccination with IIV4, HAI geometric mean titers (GMTs) had increased for each of the four strains by at least 4-fold, and seroprotection rates were at least 98.0% for all strains. Rates of seroconversion/significant increase in titer were between 39.7% (A/H1N1) and 72.4% (A/H3N2) in participants vaccinated with IIV4.

**Table 1.** HAI antibody response.

			IIV4				IIV3			
		CHMP	A/H1N1	A/H3N2	B Yamagata-lineage (B/Phuket)	B Victoria-lineage (B/Brisbane)	A/H1N1	A/H3N2	B Yamagata-lineage (B/Phuket)	B Victoria-lineage (B/Brisbane)
Measure	Day	criterion^a^	N = 199	N = 199	N = 199	N = 199	N = 99	N = 99	N = 99	N = 100
HAI GMT (95% CI)	0		142 (116, 174)	41.6 (34.1, 50.9)	256 (212, 309)	186 (156, 220)	116 (86.1, 155)	35.8 (27.2, 47.0)	219 (166, 290)	139 (107, 180)
	21		583 (516, 658)	483 (406, 574)	1068 (950, 1200)	737 (651, 835)	651 (550, 771)	691 (543, 881)	1105 (933, 1308)	290 (231, 364)
HAI titer < 10, n (%)	0		11 (5.5)	34 (17.1)	3 (1.5)	1 (0.5)	6 (6.1)	20 (20.2)	1 (1.0)	2 (2.0)
GMTR (95% CI)	21/0	> 2.5	4.1 (3.30, 5.09)	11.6 (9.16, 14.7)	4.17 (3.49, 4.98)	3.97 (3.30, 4.78)	5.64 (4.09, 7.76)	19.3 (13.9, 26.8)	5.04 (3.81, 6.67)	2.08 (1.75, 2.48)
Seroprotection^b^, % (95% CI)	0		83.4 (77.5, 88.3)	56.3 (49.1, 63.3)	91.5 (86.7, 94.9)	89.4 (84.3, 93.3)	78.8 (69.4, 86.4)	55.6 (45.2, 65.5)	87.9 (79.8, 93.6)	85.0 (76.5, 91.4)
	21	> 70%	99.5 (97.2, 100.0)	98.0 (94.9, 99.4)	100.0 (98.2, 100.0)	100.0 (98.2, 100.0)	100.0 (96.3, 100.0)	99.0 (94.5, 100.0)	100.0 (96.3, 100.0)	97.0 (91.5, 99.4)
Seroconversion or significant increase^c^	21/0	> 40%	39.7 (32.8, 46.9)	72.4 (65.6, 78.5)	48.7 (41.6, 55.9)	46.2 (39.2, 53.4)	46.5 (36.4, 56.8)	84.8 (76.2, 91.3)	53.5 (43.2, 63.6)	23.0 (15.2, 32.5)

Abbreviations: CI, confidence interval; CHMP, Committee for Medicinal Products for Human Use of the European Medicines Agency; GMT, geometric mean titer; GMTR, geometric mean of the individual ratios of the post-vaccination (day 21) HAI titer divided by the pre-vaccination (day 0) HAI titer; HAI, hemagglutination inhibition; IIV3, trivalent inactivated influenza vaccine; IIV4, quadrivalent inactivated influenza vaccine.

a According to the former CHMP Note for Guidance (CPMP/BWP/214/96)^13^.

b Seroprotection was defined as a HAI titer ≥ 40.

c Seroconversion was defined as a pre-vaccination (day 0) HAI titer < 10 and a post-vaccination (day 21) HAI titer ≥ 1:40, and a significant increase was defined as a pre-vaccination HAI titer ≥ 10 and a ≥ 4-fold increase in HAI titer.

For all strains in IIV4, geometric mean post-/pre-vaccination ratios of HAI titers were > 2.5 and seroprotection rates were > 70%. In addition, seroconversion/significant increase rates were > 40% for the A/H3N2, B Yamagata-lineage, and B Victoria-lineage strains, although not for the A/H1N1 strain, for which the rate was 39.7% (95% confidence interval [CI], 32.8–46.9). Thus, IIV4 met the requirements of the former CHMP Note for Guidance for influenza vaccines.

Immunogenicity was comparable for the three strains included in IIV3. For the additional B-lineage strain not included in IIV3 (B Victoria lineage), HAI geometric mean antibody titers were higher for IIV4 (737 [95% CI, 651–835]) than for IIV3 (290 [95% CI, 231–364]). Some cross-reactivity could be detected between the B lineage strains: 23.0% (95% CI, 15.2–32.5) of participants seroconverted or had a significant increase in titer for the missing B-lineage strain (Victoria), and the HAI titer increased from baseline by a geometric mean of 2.08 fold (95% CI, 1.75–2.48).

### Influence of vaccination the previous year on immunogenicity

At baseline, participants who were vaccinated the previous year had higher HAI GMTs and seroprotection rates than participants who were not vaccinated the previous year (Table S1). In contrast, after vaccination, participants who were vaccinated the previous year tended to have lower HAI GMTs than participants who were not vaccinated the previous year. Regardless, post-vaccination seroprotection rates for IIV4 were at least 97% for all four strains irrespective of whether the participants had been vaccinated the previous year.

### Influence of baseline serological status on immunogenicity

Because few participants had low or undetectable baseline HAI titers (<10), only the two A strains could be compared by baseline serological status (Table S2). After vaccination, participants with detectable baseline titers tended to have slightly higher HAI GMTs and seroprotection rates than participants with detectable baseline titers, although confidence intervals overlapped in most cases. However, geometric mean post-/pre-vaccination titer ratios were lower in participants who had detectable baseline titers than in participants who did not.

### Safety and reactogenicity

The most common solicited reactions to both vaccines were injection-site pain, myalgia, malaise, and headache (Table 2). Few participants (<7%) reported shivering, fever, injection-site erythema, injection-site swelling, or injection-site induration, and none reported injection-site ecchymosis. Most solicited reactions were grade 1, and few participants reported grade 3 reactions (< 2% per reaction type). All solicited reactions resolved within a maximum of 5 days. Similar proportions of participants vaccinated with the two vaccines reported solicited reactions as defined by the study protocol (Table 2) and the former CHMP Note for Guidance (Table 3).

**Table 2.** Proportions participants reporting solicited reactions.

			IIV4		IIV3	
			(N = 200)		(N = 100)	
Type	Reaction	Maximum intensity	n	% (95% CI)	n	% (95% CI)
Injection site	Pain	Any	141	70.5 (63.7, 76.7)	64	64.0 (53.8, 73.4)
		Grade 3	2	1.0 (0.1, 3.6)	0	0.0 (0.0, 3.6)
	Erythema	Any	5	2.5 (0.8, 5.7)	3	3.0 (0.6, 8.5)
		Grade 3	0	0.0 (0.0, 1.8)	0	0.0 (0.0, 3.6)
	Swelling	Any	7	3.5 (1.4, 7.1)	0	0.0 (0.0, 3.6)
		Grade 3	0	0.0 (0.0, 1.8)	0	0.0 (0.0, 3.6)
	Induration	Any	6	3 (1.1, 6.4)	2	2.0 (0.2, 7.0)
		Grade 3	0	0.0 (0.0, 1.8)	0	0.0 (0.0, 3.6)
	Ecchymosis	Any	0	0.0 (0.0, 1.8)	0	0.0 (0.0, 3.6)
		Grade 3	0	0.0 (0.0, 1.8)	0	0.0 (0.0, 3.6)
Systemic	Fever	Any	1	0.5 (0.0, 2.8)	0	0.0 (0.0, 3.6)
		Grade 3	0	0 (0.0, 1.8)	0	0.0 (0.0, 3.6)
	Headache	Any	33	16.5 (11.6, 22.4)	20	20.0 (12.7, 29.2)
		Grade 3	3	1.5 (0.3, 4.3)	2	2.0 (0.2, 7.0)
	Malaise	Any	67	33.5 (27.0, 40.5)	42	42.0 (32.2, 52.3)
		Grade 3	3	1.5 (0.3, 4.3)	1	1.0 (0.0, 5.4)
	Myalgia	Any	102	51.0 (43.9, 58.1)	49	49 (38.9, 59.2)
		Grade 3	3	1.5 (0.3, 4.3)	0	0.0 (0.0, 3.6)
	Shivering	Any	13	6.5 (3.5, 10.9)	6	6.0 (2.2, 12.6)
		Grade 3	0	0 (0.0, 1.8)	0	0.0 (0.0, 3.6)

Solicited reactions were recorded by participants up to 7 days after vaccination on diary cards. Erythema, swelling, induration, and ecchymosis were considered grade 1 for ≥ 25 to ≤ 50 mm, grade 2 for ≥ 51 to ≤ 100 mm, and grade 3 for > 100 mm. Fever was considered grade 1 for ≥ 38.0°C to ≤ 38.4°C, grade 2 for ≥ 38.5°C to ≤ 38.9°C, and grade 3 for ≥ 39.0°C. All other reactions and AEs were considered grade 1 for not interfering with activity, grade 2 for some interference with activity, and grade 3 for significant, preventing daily activity. Abbreviations: CI, confidence interval; IIV3, trivalent inactivated influenza vaccine; IIV4, quadrivalent inactivated influenza vaccine.

**Table 3.** Solicited reactions in the former CHMP Note for Guidance^a^ occurring within 3 days of vaccination.

	IIV4		IIV3	
	(N = 200)		(N = 100)	
Reaction	n	% (95% CI)	n	% (95% CI)
Injection-site induration ≥ 50 mm for ≥ 4 consecutive days	0	0.0 (0.0; 1.8)	0	0.0 (0.0; 3.6)
Injection-site ecchymosis	12	6 (3.1; 10.2)	4	4.0 (1.1; 9.9)
Pyrexia (temperature > 38.0°C) for ≥ 24 h	0	0.0 (0.0; 1.8)	0	0.0 (0.0; 3.6)
Malaise	62	31.0 (24.7; 37.9)	40	40.0 (30.3; 50.3)
Shivering	11	5.5 (2.8; 9.6)	6	6.0 (2.2; 12.6)

Abbreviations: CHMP, Committee for Human Vaccines and Medical Products; CI, confidence interval; IIV3, trivalent inactivated influenza vaccine; IIV4, quadrivalent inactivated influenza vaccine.

a Former CHMP Note for Guidance (CPMP/BWP/214/96)^13^.

Adverse events (AEs) considered to be related to vaccination were recorded for two participants vaccinated with IIV4, both of whom had moderate vaginal hemorrhage. No immediate AEs, serious adverse events (SAEs), adverse events of special interest (AESIs), or AEs leading to discontinuation were reported.

## Discussion

This study, performed in the Republic of Korea, showed that the 2015–2016 Northern Hemisphere formulation of IIV4 was highly immunogenic and well tolerated in adults 18–60 years of age. Although most participants in this study had high pre-existing antibodies, HAI titers increased, on average, by at least 4-fold for each of the four vaccine strains in IIV4. In addition, IIV4 appeared safe, with no new safety signals and no obvious differences in reactogenicity compared to IIV3.

IIV4 was evaluated in this study according to the former CHMP Note for Guidance^13^ in agreement with the Republic of Korea Ministry of Food and Drug Safety. IIV4 met the requirements for influenza vaccines, although the seroconversion rate for the A/H1N1 strain was just under the 40% limit. This was likely due to high baseline antibody titers as a result of previous vaccinations or natural exposure. Because nearly all participants had pre-existing antibodies, we were not able to explore the impact of baseline titers for B strains; however, previous vaccination resulted in higher baseline titers and lower post-vaccination titers. We also found that post-vaccination immunogenicity tended to be slightly lower in participants with pre-existing antibodies, but because nearly all participants had pre-existing antibodies, we could not reliably assess their impact. Irrespective of baseline serological or vaccination status, responses to IIV4 were considered satisfactory.

In children, adolescents, and adults, IIV4 induces non-inferior HAI antibody responses compared to IIV3 for the three shared strains and a superior response for the additional B strain lineage.^10-12^ Similarly, in children, adolescents, and adults, the quadrivalent live attenuated influenza vaccine induces non-inferior HAI antibody responses compared to the licensed trivalent live attenuated vaccine for the three shared strains, although superiority for the alternate B strain has not been reported.^14,15^ In this study, immunogenicity was comparable between IIV4 and IIV3 for the three shared strains of influenza and appeared higher for IIV4 than for IIV3 for the additional B strain lineage in IIV4 (B Victoria). Therefore, although we did not perform a statistical analysis of non-inferiority or superiority, the current results appear to be in line with those from the other studies.

IIV3 appeared to induce some cross-reactivity against the missing B-Victoria lineage strain. Low-level B-strain cross-reactivity has been previously documented in adults.^11,16^ However, the cross-reactivity found here and in these other studies was much weaker than the immune response induced by IIV4. The study also showed that vaccination the previous year tended to reduce the antibody response to the influenza vaccine. Similarly, having pre-existing antibodies appeared to slightly, but not significantly, reduce lower the antibody response. In all cases, however, the vaccine was highly immunogenic.

The study was limited by the relatively small sample size, which allowed reliable detection of only common AEs (occurring in > 1% of the study population). In addition, because this was a confirmatory study, a prospective statistical analysis was not included, and the population size (300) and randomization ratio (2:1) were arbitrary. This precludes drawing meaningful conclusions from the statistical analyses, although the results were in line with the other studies specifically designed to assess non-inferiority and superiority of immune responses to IIV4 vs. IIV3.

In summary, this study suggested that IIV4 is safe and induces adequate antibody responses in Korean individuals. The results also confirm previous findings that including a second B strain lineage should improve protection against B-strain influenza without affecting vaccine safety and without affecting protection against the other three vaccine strains. These results add to the limited data that have been published on the immunogenicity and safety influenza vaccines in Asian populations, B cross-lineage immunogenicity, and the influence of pre-existing antibodies and vaccination the previous year on influenza vaccine immunogenicity.

## Patients and methods

### Study design

This was an observer-blind, randomized, controlled trial (ClinicalTrials.gov no. NCT02550197) conducted at five centers in the Republic of Korea. The objectives of this study were to evaluate the immunogenicity and safety of the IIV4 and IIV3 Northern Hemisphere 2015–2016 formulations and the compliance of IIV4 with the requirements of the CHMP former Note for Guidance CPMP/BWP/214/96^13^ as requested by the Republic of Korea Ministry of Food and Drug Safety.

### Ethics

The study was approved by each institution's ethics committee or review board. The conduct of this trial was consistent with the standards established by the Declaration of Helsinki and complied with the International Conference on Harmonization Guidelines for Good Clinical Practice as well as all local and national regulations and directives. All participants provided written informed consent to be included in this trial.

### Participants

The study was planned to include 300 adults 18–60 years of age. Participants could not have received any vaccine in the 4 weeks preceding the trial vaccination or have planned receipt of any vaccine during the study; been vaccinated against influenza or self-reported influenza infection or influenza-like illness in the previous 6 months; received immune globulins, blood, or blood-derived products in the past 3 months; be immunodeficient or taking immunosuppressive therapy; have a history of seropositivity for human immunodeficiency virus or hepatitis C; be hypersensitive to eggs, chicken proteins, or any vaccine components; have a bleeding disorder or receipt of anticoagulants in the 3 weeks before inclusion or known or suspected thrombocytopenia contraindicating intramuscular vaccination; have febrile illness (temperature ≥ 38.0°C) on the day of vaccination; or have any illness that could interfere with trial conduct or completion. Women could not be pregnant or lactating and, if of childbearing potential, had to be abstinent or using an effective method of contraception from at least 4 weeks prior to vaccination and until at least 3 weeks after vaccination.

### Vaccines

As recommended by the World Health Organization and the European Union for the 2015–2016 Northern Hemisphere influenza season, each 0.5-ml dose of IIV4 (Sanofi Pasteur) contained 15 µg of hemagglutinin from the A/California/7/2009 (H1N1), A/South Australia/55/2014 (H3N2), B/Phuket/3073/2013 (Yamagata lineage), and B/Brisbane/60/2008 (Victoria lineage) viruses. Each 0.5-ml dose of IIV3 (Vaxigrip, Sanofi Pasteur) contained 15 µg of hemagglutinin of the same A/H1N1, A/H3N2, and B Yamagata-lineage viruses. Both vaccines were thimerosal-free, inactivated, split-virion formulations.

### Study conduct

Participants were randomly assigned in a 2:1 ratio to receive one 0.5-ml dose of IIV4 or IIV3 by intramuscular injection into the deltoid muscle. Blood samples were taken just before the vaccine was administered (day 0) and 21 ± 3 days after vaccination. Participants were followed-up for safety for 21 days after vaccination.

### Assessment of immunogenicity

The primary immunogenicity endpoint was the HAI titer measured 21 days post-vaccination. HAI titers were also assessed at baseline (day 0). HAI titers were measured as described previously^10^ at a central location (Sanofi Pasteur, Swiftwater, PA, USA). Briefly, the highest serum dilution resulting in complete inhibition of hemagglutination was determined for duplicates of each sample. The HAI antibody titer for each sample was calculated as the geometric mean of the reciprocal of the duplicate values. The lower limit of quantitation was set at the reciprocal of the lowest dilution used in the assay (10), and the upper limit of quantitation as the highest dilution used in the assay (10,240). GMTs, geometric means of the individual titer ratio (day 21 vs. day 0), seroprotection, and seroconversion/significant increase in HAI titer were calculated. Seroprotection was defined as a HAI titer ≥ 40, seroconversion as a HAI titer < 10 on day 0 and a post-vaccination HAI titer ≥ 40, and significant increase was defined as a HAI titer ≥ 10 on day 0 and a ≥ 4-fold post-vaccination increase in HAI titer.

### Safety and reactogenicity

Unsolicited AEs and SAEs were collected according to the International Committee for Harmonization E2A Guideline for Clinical Safety Data Management: Definitions and Standards for Expedited Reporting.^17^ Investigators recorded unsolicited AEs, SAEs and AESIs occurring within 21 days. AESIs included anaphylaxis, Guillain-Barré syndrome, encephalitis/myelitis, neuritis, febrile and non-febrile convulsions, thrombocytopenia, and vasculitis. Investigators categorized the relatedness of all unsolicited AEs, SAEs, and AESIs as “not related” or as “related”.

Participants recorded on diary cards solicited injection-site reactions (pain, erythema, swelling, induration, ecchymosis) and solicited systemic reactions (fever, headache, malaise, myalgia, shivering) occurring within 7 days. Erythema, swelling, induration, and ecchymosis were considered grade 1 for ≥ 25 to ≤ 50 mm, grade 2 for ≥ 51 to ≤ 100 mm, and grade 3 for > 100 mm. Fever was considered grade 1 for ≥ 38.0°C to ≤ 38.4°C, grade 2 for ≥ 38.5°C to ≤ 38.9°C, and grade 3 for ≥ 39.0°C. All other reactions were considered grade 1 for not interfering with activity, grade 2 for some interference with activity, and grade 3 for significant, preventing daily activity. In addition, reactions listed in the former CHMP Note for Guidance occurring within 3 days are reported, including injection site induration ≥ 50 mm and persisting for at least 4 consecutive days, injection site ecchymosis, temperature > 38°C for ≥ 24 h, malaise, and shivering.

### Statistical analysis

Statistical analysis was performed using SAS® version 9.4 (SAS Institute, Cary, NC, USA). Missing or incomplete data were not replaced, with the exception that all HAI titers under the lower limit of quantitation (10) were assigned a value of 5 and all HAI titers above the upper limit of quantitation (10,240) were assigned a value of 10,240. Immunogenicity was assessed in all participants who had received one dose of the study vaccines as randomized, who had pre- and post-vaccination titers available, and who met the selection criteria. To calculate GMTs, the means and 95% CIs were determined from log_10_-transformed data using Student's t-distribution with n−1 degrees of freedom, after which antilog transformations were applied to the results of calculations. Safety was assessed in all participants according to the vaccine they received. Compliance with former CHMP Note for Guidance^13^ for adults 18–60 years of age was assessed and included at least one of the following: a rate of seroconversion or significant increase of titer 21 days after vaccination > 40%; a mean geometric increase from pre- to post-vaccination titers > 2.5; or a rate of seroprotection 21 days after vaccination > 70%. No formula was used to compute the sample size because no statistical comparisons were made.

## Supplementary Material

Supplemental_Material.pdf
