# Supplementary material for: Immunogenicity and safety of a split-virion quadrivalent influenza vaccine in adults 18–60 years of age in the Republic of Korea
Source: Hum Vaccin Immunother. 2017 Nov 17;14(3):587–92. doi: 10.1080/21645515.2017.1381808 (PMC5861787; doi:10.1080/21645515.2017.1381808)
Supplement: Supplemental_Material.pdf [file khvi-14-03-1381808-s001.pdf]

Supplemental online information

Immunogenicity and safety of a split-virion quadrivalent influenza vaccine in adults 18–60 years of age in the Republic of Korea

Table S1. Immunogenicity by previous year vaccination status

			IIV4				IIV3			
Day	Measure	Vaccinated the previous year	A/H1N1	A/H3N2	B Yamagata-lineage (B/Phuket)	B Victoria-lineage (B/Brisbane)	A/H1N1	A/H3N2	B Yamagata-lineage (B/Phuket)	B Victoria-lineage (B/Brisbane)
-	N	Yes	85	85	85	85	33	33	33	33
		No	113	113	113	113	66	66	66	67
0	HAI GMT (95% CI)	Yes	242 (187, 311)	60.1 (45.5, 79.4)	359 (279, 462)	300 (237, 380)	191 (124, 295)	47.3 (31.5, 71.1)	249 (154, 403)	229 (146, 359)
		No	96.2 (72.2, 128)	31.2 (23.7, 41.2)	200 (154, 261)	129 (103, 162)	89.8 (61.6, 131)	31.1 (21.8, 44.4)	206 (145, 293)	109 (80.2, 148)
	Seroprotection[a], % (95% CI)	Yes	94.1 (86.8, 98.1)	68.2 (57.2, 77.9)	98.8 (93.6, 100.0)	96.5 (90.0, 99.3)	84.8 (68.1, 94.9)	66.7 (48.2, 82.0)	93.9 (79.8, 99.3)	90.9 (75.7, 98.1)
		No	75.2 (66.2, 82.9)	46.9 (37.5, 56.5)	85.8 (78.0, 91.7)	84.1 (76.0, 90.3)	75.8 (63.6, 85.5)	50.0 (37.4, 62.6)	84.8 (73.9, 92.5)	82.1 (70.8, 90.4)
21	HAI GMT (95% CI)	Yes	417 (346, 503)	405 (320, 513)	890 (751, 1056)	643 (532, 777)	429 (343, 537)	564 (406, 784)	711 (527, 958)	288 (188, 441)
		No	762 (661, 879)	551 (429, 707)	1226 (1046, 1437)	823 (697, 972)	802 (649, 992)	765 (552, 1061)	1378 (1142, 1662)	292 (222, 384)
	Seroprotection[a], % (95% CI)	Yes	98.8 (93.6, 100.0)	98.8 (93.6, 100.0)	100.0 (95.8, 100.0)	100.0 (95.8, 100.0)	100.0 (89.4, 100.0)	100.0 (89.4, 100.0)	100.0 (89.4, 100.0)	93.9 (79.8, 99.3)
		No	100.0 (96.8, 100.0)	97.3 (92.4, 99.4)	100.0 (96.8, 100.0)	100.0 (96.8, 100.0)	100.0 (94.6, 100.0)	98.5 (91.8, 100.0)	100.0 (94.6, 100.0)	98.5 (91.8, 100.0)
21/0	GMTR (95% CI)	Yes	1.73 (1.48, 2.02)	6.74 (4.93, 9.21)	2.48 (2.05, 3.01)	2.14 (1.75, 2.62)	2.24 (1.60, 3.16)	11.9 (7.42, 19.2)	2.86 (1.93, 4.23)	1.26 (1.06, 1.50)
		No	7.93 (5.79, 10.9)	17.6 (12.7, 24.4)	6.13 (4.74, 7.93)	6.36 (4.90, 8.25)	8.93 (5.93, 13.5)	24.6 (16.1, 37.7)	6.69 (4.68, 9.57)	2.67 (2.14, 3.34)

Abbreviations: CI, confidence interval; GMT, geometric mean titer; GMTR, geometric mean of the individual ratios of the post-vaccination (day 21) HAI titer divided by the pre-vaccination (day 0) HAI titer; HAI, hemagglutination inhibition; IIV3, trivalent inactivated influenza vaccine; IIV4, quadrivalent inactivated influenza vaccine; NC, not calculable.

[a] Seroprotection was defined as a HAI titer ≥ 40

Table S2. Immunogenicity by baseline serological status

Day	Measure	Baseline HAI titer	IIV4 A/H1N1	IIV4 A/H3N2	IIV4 B Yamagata-lineage (B/Phuket)	IIV4 B Victoria-lineage (B/Brisbane)	IIV3 A/H1N1	IIV3 A/H3N2	IIV3 B Yamagata-lineage (B/Phuket)	IIV3 B Victoria-lineage (B/Brisbane)
-	N	< 10	11	34	3	1	6	20	1	2
		≥ 10	188	165	196	198	93	79	98	98
0	HAI GMT (95% CI)	< 10	5.33 (4.85, 5.85)	5.42 (5.15, 5.71)	7.07 (NC)	5.00 (NC)	5.30 (4.57, 6.15)	5.27 (4.96, 5.59)	7.07 (NC)	7.07(NC)
		≥ 10	172 (144, 206)	63.4 (52.6, 76.3)	271 (226, 324)	189 (160, 224)	141 (108, 184)	58.1 (45.6, 73.9)	227 (173, 299)	148 (115, 190)
	Seroprotection[a], % (95% CI)	< 10	0.0 (0.0, 8.5)	0.0 (0.0, 10.3)	0.0 (0.0, 0.8)	0.0 (0.0, 97.5)	0.0 (0.0, 5.9)	0.0 (0.0, 6.8)	0.0 (0.0, 7.5)	0.0 (0.0, 84.2)
		≥ 10	88.3 (82.8, 92.5)	67.9 (60.2, 74.9)	92.9 (88.3, 96.0)	89.9 (84.8, 93.7)	83.9 (74.8, 90.7)	69.6 (58.2, 79.5)	88.8 (80.8, 94.3)	86.7 (78.4, 92.7)
21	HAI GMT (95% CI)	< 10	530 (212, 1324)	258 (155, 431)	359 (NC)	320 (NC)	479 (150, 1529)	422 (203, 878)	453 (NC)	95.1 (NC)
		≥ 10	586 (519, 661)	549 (460, 656)	1086 (967, 1220)	740 (653, 839)	664 (560, 788)	783 (614, 999)	1115 (941, 1321)	297 (237, 373)
	Seroprotection[a], % (95% CI)	< 10	90.9 (58.7, 99.8)	91.2 (76.3, 98.1)	100.0 (29.2, 100.0)	100.0 (2.5, 100.0)	100.0 (54.1, 100.0)	95.0 (75.1, 99.9)	100.0 (2.5, 100.0)	50.0 (1.3, 98.7)
		≥ 10	100.0 (98.1, 100.0)	99.4 (96.7, 100.0)	100.0 (98.1, 100.0)	100.0 (98.1, 100.0)	100.0 (96.1, 100.0)	100.0 (96.1, 100.0)	100.0 (96.1, 100.0)	98.0 (92.8, 99.8)

Abbreviations: CI, confidence interval; GMT, geometric mean titer; GMTR, geometric mean of the individual ratios of the post-vaccination (day 21) HAI titer divided by the pre-vaccination (day 0) HAI titer; HAI, hemagglutination inhibition; IIV3, trivalent inactivated influenza vaccine; IIV4, quadrivalent inactivated influenza vaccine.

[a] Seroprotection was defined as a HAI titer ≥ 40
